# Effects of Incentive-Based Smoking Cessation Program for Pregnant Women on Birth Outcomes

**DOI:** 10.1007/s10995-016-2166-y

**Published:** 2016-07-29

**Authors:** Xianglan Zhang, Rose Devasia, Glenn Czarnecki, Jan Frechette, Sarah Russell, Bruce Behringer

**Affiliations:** 1 Tennessee Department of Health, Andrew Johnson Tower, 2nd Floor, 710 James Robertson Parkway, Nashville, TN 37243 USA; 2 Tennessee Department of Health, Andrew Johnson Tower, 8th Floor, 710 James Robertson Parkway, Nashville, TN 37243 USA; 3 Tennessee Department of Health, Southeast Regional Office, 1301 Riverfront Parkway, Suite 209, Chattanooga, TN 37402 USA; 4 Tennessee Department of Health, South Central Regional Office, 1216 Trotwood Avenue, Columbia, TN 38401 USA; 5 Tennessee Department of Health, Andrew Johnson Tower, 5th Floor, 710 James Robertson Parkway, Nashville, TN 37243 USA

**Keywords:** Incentives, Smoking cessation, Pregnancy, Birth outcomes

## Abstract

*Objectives* Cigarette smoking during pregnancy is an important modifiable risk factor for poor birth outcomes. We evaluated whether participation in a statewide incentive-based smoking cessation program for pregnant women, the Baby & Me-Tobacco Free (BMTF) program, was associated with improved birth outcomes. *Methods* Linked program and birth certificate data from 866 pregnant smokers who participated in the BMTF program and 11,568 pregnant smokers who were eligible for but did not enroll in the program were analyzed. The BMTF program consisted of 4 prenatal smoking cessation counselling sessions, 12 postpartum follow-up visits, breath carbon monoxide measurements to monitor smoking status, and rewards of diaper vouchers for quitting smoking. Logistic regression models were used to examine the associations of program participation with infant low birth weight and preterm birth. *Results* Participants who completed 3–4 prenatal smoking cessation sessions had a significantly lower rate of low birth weight than non-participants (4.9 vs. 11.6 %). After adjustment for multiple potential confounders, the odds ratios for low birth weight were 0.51 (95 % confidence interval, 0.30–0.88) in those participants completing 3–4 sessions and 0.37 (95 % confidence interval, 0.17–0.79) in participants who quit smoking, as compared with non-participants. Although not statistically significant, a protective effect was also suggested for preterm birth. *Conclusions* We found for the first time that successful participation in the BMTF program, a unique incentive-based smoking cessation program for pregnant women implemented in community settings, was associated with significantly reduced odds of having a low birth weight infant.

## Significance

*What is already known on this subject?* Although use of financial incentives has been shown to increase smoking cessation in pregnant smokers in randomized controlled trials, data are lacking on the effectiveness of incentive-based smoking cessation programs for pregnant women implemented in “real” world community settings. Moreover, the effects of such programs on birth outcomes remain unclear.

*What this study adds?* This study provides the first evidence that successful participation in a unique statewide incentive-based smoking cessation program tailored to pregnant women, the Baby & Me-Tobacco Free program, was associated with improved birth outcomes.

## Introduction

Cigarette smoking during pregnancy is a major modifiable risk factor for poor birth outcomes, including low birth weight, preterm birth, and sudden infant death syndrome (U.S. Department of Health and Human Services 2014). Despite these and many other neonatal and maternal health consequences of smoking during pregnancy, the prevalence of smoking during pregnancy remains high in the United States (Tong et al. 2013). The prevalence, however, varies among states. Data from national, population-based surveys have shown that the prevalence of smoking during pregnancy in Tennessee was considerably higher than the national average (22.2 vs. 12.4 % in 2009; Tong et al. 2013). Whereas the percentage of smokers who quit smoking during pregnancy in 2009 was 35.7 % in Tennessee, nationally it was 51.7 % (Tong et al. 2013). According to recent National Vital Statistics Reports based on 2014 birth certificate data, the prevalence of smoking during pregnancy was 14.9 in Tennessee and 8.4 in the total reporting area including 46 states and District of Columbia (Curtin and Mathews 2016). The rate of quitting during pregnancy was 21.2 in Tennessee and 20.6 in the reporting area (Curtin and Mathews 2016). These data indicate that smoking during pregnancy remains a significant public health challenge in Tennessee and underscores the need for identifying more effective strategies to address this challenge.

There is growing evidence supporting the effectiveness of financial incentive-based approaches for health behavior changes including smoking cessation (Volpp et al. 2009; Tappin et al. 2015; Giles et al. 2014). In an effort to address the burden of smoking during pregnancy in Tennessee, the Tennessee Department of Health adopted an innovative, incentive-based cessation program tailored to pregnant women, the Baby & Me-Tobacco Free (BMTF) program. In addition to free cessation counselling, the BMTF program provides free diaper vouchers as a tangible incentive to motivate pregnant smokers to quit smoking. Studies have shown a potential link between diaper need and increasing parental stress and worsening child health (Smith et al. 2013), suggesting that using free diaper vouchers as an incentive could provide additional health benefits.

In recent years, the BMTF program has gained national recognition. Although the program has been shown to be effective in promoting smoking cessation in pregnant women (Gadomski et al. 2011), no study has evaluated whether program participation is associated with better birth outcomes. In the present study, we examined the associations of program participation with infant low birth weight and preterm birth by using data from the Tennessee BMTF program and birth certificate data.

## Methods

### Study Population

The Tennessee BMTF program, supported by tobacco Master Settlement Agreement funds, was launched in April 2014. Funding for projects aimed at reducing smoking in pregnancy was distributed to all counties. Of 95 counties, 86 chose to implement the BMTF program. To most efficiently reach a large number of pregnant women who smoke, the program was set up in local health department clinics and integrated with existing public health services, such as the Special Supplemental Nutrition Program for Women, Infants, and Children (WIC). All pregnant women who were current smokers, regardless of their income level, were invited to participate in the BMTF program via flyers and informational sessions at the local health departments and community settings. Program staff underwent structured training, which emphasized the 5A’s approach (ask about smoking, advise to quit, assess willingness, assist in quitting, and arrange follow-up) and motivational interviewing strategies (Clinical Practice Guideline Treating Tobacco Use and Dependence 2008 Update Panel, Liaisons, and Staff 2008). The program provided 4 prenatal smoking cessation classes, where participants received face-to-face counselling on smoking cessation and support. In addition, participants were tested for breath carbon monoxide (CO) levels as a measure of smoking status (Middleton and Morice 2000). Participants were also asked to attend monthly postpartum follow-up visits and take breath CO tests; they were awarded a $25 voucher for diapers each month for up to 12 months if their postpartum CO testing results were within normal ranges. As of June 30, 2015, 1950 women enrolled in the program. For this study, we included women who had a single live birth by 30 November 2015 and for whom the BMTF program records were linked to child’s birth certificate data (n = 869). By using birth certificate data, we also identified, as a comparison group, a cohort of pregnant women who reported smoking during pregnancy, had a single live birth during the BMTF program period, were residents of the program participating counties, and were eligible for but did not enroll in the program (n = 11,634). Possible reasons for non-enrollment included the inability to reach all potential participants at the local health departments and among those approached for participation, a lack of willingness, readiness, or confidence to quit smoking, fear of weight gain and increased stress associated with quitting, a lack of interest in the incentives, and a lack of time or transportation. This study was conducted in accord with prevailing ethical principles and approved by the Institutional Review Board of Tennessee Department of Health.

### Assessment of Breath CO Levels and Smoking Cessation

Trained staff at the local health department clinics performed breath CO testing using a breath CO monitor. A breath CO reading of ≤6 parts per million (ppm) was classified as normal (non-smoker) range (Middleton and Morice 2000). Smoking cessation during pregnancy for this study was defined as having a breath CO reading within the normal range at more than 2 out of 4 total prenatal visits.

### Assessment of Birth Outcomes and Covariates

The Tennessee Vital Statistics System collects birth data using the U.S. standard certificate of live birth form (Hamilton and Martin 2015). Birth data includes information on maternal age, marital status, race, Hispanic origin, education, health insurance, prenatal care usage, WIC program participation, maternal cigarette smoking (the average number of cigarettes smoked per day during the 3 months before pregnancy and each trimester of pregnancy), pre-pregnancy body mass index (calculated by dividing weight in kilograms by the square of height in meters), history of pre-gestational and gestational diabetes and hypertension, period of gestation, infant’s birth weight, and other maternal and infant’s medical and health information (Hamilton and Martin 2015). In addition, household income, which is not listed on the U.S. standard birth certificate, is also collected in Tennessee.

The primary outcomes for the present study were infant low birth weight and preterm birth. Low birth weight was defined as a birth weight of less than 2500 g. Preterm birth was defined as a birth of less than 37 weeks of gestational age based on the obstetric estimate of gestation at delivery.

### Statistical Analysis

We excluded 69 women with missing data on gestational age or infant birth weight, leaving 12,434 pregnant smokers for final analyses (including 866 BMTF program participants and 11,568 non-participants). We compared the distribution of sociodemographic characteristics, public health and medical service utilization, history of cigarette smoking and other risk factors for adverse birth outcomes, and birth outcomes between BMTF participants and non-participants. *T* tests and ANOVA were used in the analysis of continuous variables and Chi square tests in the analysis of categorical variables. Logistic regression models were used to calculate odds ratios (ORs) and 95 % confidence intervals (CIs) of low birth weight and preterm birth associated with the BMTF program participation, and to adjust for potential confounding factors. Women were classified into 3 groups: non-participants (defined as pregnant smokers who were eligible for but not enrolled in the BMTF program), participants with low prenatal session attendance (defined as those who enrolled but attended only 1–2 prenatal smoking cessation sessions), and participants with high prenatal session attendance (defined as those who attended 3–4 cessation sessions); the non-participation group served as the reference group. Multivariable analyses were stratified by county of residence and adjusted for maternal age (4 categories), marital status (yes/no), race/ethnicity (4 categories), education level (3 categories), annual household income (3 categories), health insurance (3 categories), number of prenatal care visits (4 categories), WIC participation status (yes/no), pre-pregnancy body mass index (4 categories), and history of pre-gestational or gestational diabetes or hypertension (yes/no). To further evaluate the effects of program engagement and smoking cessation during pregnancy on birth outcomes, we also divided women into non-participants, BMTF participants with no evidence of quitting smoking, and BMTF participants with evidence of quitting smoking on the basis of breath CO measurement. We examined the associations of program engagement and smoking cessation during pregnancy with low birth weight and preterm birth, while adjusting for multiple potential confounders. In addition, we conducted analyses stratified by prior parity status (nulliparous/parous) to examine potential effect modification. Statistical analyses were performed by using SAS statistical software (version 9.3; SAS Institute Inc, Cary, NC). All statistical tests were based on 2-sided probability.

## Results

Table 1 presents the distribution of selected characteristics of the study population according to BMTF program participation. Compared with non-participants, BMTF participants were younger when giving birth, had a higher proportion of teen birth, and were less likely to be married, to be non-Hispanic black, and to have a high education or high income. They were more likely to be enrolled in the state Medicaid insurance and WIC programs, and had more prenatal care visits and a higher pre-pregnancy body mass index. No difference was found in the occurrence of pre-gestational or gestational diabetes or hypertension between participants and non-participants.

**Table 1:** Selected characteristics of pregnant smokers according to BMTF^a^ program participation

Variables	Non-Participants (n = 11,568)	BMTF participants (n = 866)	*P* value
Age at birth (years), mean (SD^b^)	25.8 (5.2)	24.9 (5.1)	<0.0001
Teen birth (%)	8.6	12.3	0.0003
Unmarried (%)	66.0	73.5	<0.0001
Maternal race/ethnicity (%)			
Non-Hispanic white	84.1	86.5	
Non-Hispanic black	11.3	6.6	
Hispanic	3.9	7.0	
Others	0.7	0.0	0.01
Education (%)			
High school graduate or less	69.4	67.3	
Some college or associate’s degree	28.2	31.2	
Bachelor’s degree or higher	2.4	1.5	0.05
Annual household income ($) (%)			
<15,000	56.1	56.2	
15,000–49,999	37.2	40.8	
≥50,000	6.7	3.1	0.0005
Medicaid/Tenncare (%)	80.4	87.0	<0.0001
WIC^c^ participation (%)	68.5	93.4	<0.0001
No. of prenatal care visit, mean (SD)	10.2 (4.4)	11.1 (3.8)	<0.0001
Pre-pregnancy body mass index, mean (SD)	26.3 (7.0)	27.3 (8.3)	0.0004
Pre-gestational or gestational diabetes (%)	6.4	5.0	0.10
Pre-gestational or gestational hypertension (%)	6.7	8.0	0.16

^a^BMTF denotes baby & me-tobacco free

^b^SD denotes standard deviation

^c^WIC denotes women, infants, and children

On average, non-participants smoked 17.9 cigarettes per day in the 3 months before pregnancy, and 12.9, 9.6, and 8.4 cigarettes per day during the first, second and third trimesters, respectively (Table 2). The mean number of cigarettes smoked daily in the 3 months before pregnancy or during the first trimester did not differ by BMTF program participation. However, during the second and third trimesters, BMTF participants who attended 3–4 prenatal cessation sessions smoked significantly fewer cigarettes per day than did non-participants and participants with low session attendance. The mean number of cigarettes smoked per day during the last trimester was 3.1 in participants with high session attendance versus 8.4 in non-participants. Participants with high session attendance also appeared to have lower rates of low birth weight and preterm birth compared with non-participants and participants with low session attendance (Table 2). The percentage of low birth weight infants was 4.9 % in participants with high session attendance versus 11.6 % in non-participants. On the basis of breath CO measurement, the rate of smoking cessation during pregnancy was 68.3 % in participants with high session attendance.

**Table 2:** Cigarette consumption of pregnant smokers before and during pregnancy and birth outcomes according to BMTF^a^ prenatal session attendance

Variables	Non-Participants	BMTF participants		*P* value
	(n = 11,568)	Low attendance (n = 557)	High attendance (n = 309)	
No. of cigarettes smoked/day, mean (SD^b^)				
3 months before pregnancy	17.9 (13.7)	19.2 (14.7)	17.9 (9.6)	0.15
First trimester	12.9 (11.1)	13.5 (11.0)	13.0 (7.6)	0.53
Second trimester	9.6 (10.5)	9.6 (7.4)	5.4 (6.2)	<0.0001
Third trimester	8.4 (9.7)	7.7 (6.8)	3.1 (4.7)	<0.0001
Birth outcomes, No. (%)				
Low birth weight	1339 (11.6)	61 (11.0)	15 (4.9)	0.001
Preterm birth	1347 (11.6)	73 (13.1)	25 (8.1)	0.08

^a^BMTF denotes baby & me-tobacco free

^b^SD denotes standard deviation

ORs and 95 % CIs of low birth weight and preterm birth according to BMTF program participation and smoking cessation during pregnancy are summarized in Tables 3 and 4. Participants with high session attendance had an OR of 0.39 (95 % CI 0.23–0.66) for low birth weight and an OR of 0.67 (95 % 0.44–1.01) for preterm birth, as compared with non-participants. The ORs comparing participants who quit smoking versus non-participants were 0.26 (95 % CI 0.12–0.56) for low birth weight and 0.54 (95 % CI 0.31–0.93) for preterm birth. These protective associations remained statistically significant for low birth weight but not for preterm birth after adjusting for multiple potential confounders. The strengths of the associations, however, were attenuated. No significant associations were found for participants with low session attendance or who had no evidence of quitting smoking on the basis of breath CO measurement.

**Table 3:** Odds ratios (ORs) and 95 % confidence intervals (CIs) of low birth weight in pregnant smokers according to BMTF^a^ prenatal session attendance and smoking cessation

Variables	Crude OR (95 % CI)	Multivariable-adjusted OR^b^ (95 % CI)
Program participation		
Non-participants	Referent	Referent
Participants with low attendance	0.94 (0.72–1.23)	1.02 (0.77–1.36)
Participants with high attendance	0.39 (0.23–0.66)	0.51 (0.30–0.88)
Smoking cessation		
Non-participants	Referent	Referent
Participants without evidence of quitting	0.90 (0.70–1.16)	0.99 (0.76–1.30)
Participants with evidence of quitting	0.26 (0.12–0.56)	0.37 (0.17–0.79)

^a^BMTF denotes baby & me-tobacco free

^b^Multivariable-adjusted OR: stratified by county of residence and adjusted for age, marital status, race/ethnicity, education, household income, insurance, number of prenatal care visit, WIC participation status, pre-pregnancy body mass index, history of pre-gestational diabetes or hypertension, and gestational diabetes or hypertension

**Table 4:** Odds ratios (ORs) and 95 % confidence intervals (CIs) of preterm birth in pregnant smokers according to BMTF^a^ prenatal session attendance and smoking cessation

Variables	Crude OR (95 % CI)	Multivariable-adjusted OR^b^ (95 % CI)
Program participation		
Non-participants	Referent	Referent
Participants with low attendance	1.14 (0.89–1.47)	1.23 (0.94–1.62)
Participants with high attendance	0.67 (0.44–1.01)	0.81 (0.52–1.26)
Smoking cessation		
Non-participants	Referent	Referent
Participants without evidence of quitting	1.12 (0.88–1.41)	1.21 (0.94–1.57)
Participants with evidence of quitting	0.54 (0.31–0.93)	0.68 (0.39–1.20)

^a^BMTF denotes baby & me-tobacco free

^b^Multivariable-adjusted OR: stratified by county of residence and adjusted for age, marital status, race/ethnicity, education, household income, insurance, number of prenatal care visit, WIC participation status, pre-pregnancy body mass index, history of pre-gestational diabetes or hypertension, and gestational diabetes or hypertension

In stratified analyses, we found no statistically significant effect modification of prior parity status on the associations between smoking cessation and birth outcomes. The ORs of low birth weight for participants who quit smoking versus non-participants were 0.25 (95 % CI 0.08–0.79) and 0.27 (95 % CI 0.10–0.75) among women with and without prior live births, respectively (*P* for interaction = 0.62). The corresponding ORs for preterm birth were 0.57 (95 % CI 0.26–1.24) and 0.55 (95 % CI 0.26–1.20) among women with and without prior live births, respectively (*P* for interaction = 0.51).

## Discussion

In this study, we found that completion of 3–4 prenatal smoking cessation sessions offered by the BMTF program was associated with significantly reduced odds of having a low birth weight infant. This association persisted after adjustment for a range of potential confounding variables. Our study provides the first evidence that the BMTF program, a unique incentive-based, statewide smoking cessation program for pregnant women implemented in community settings, was effective at reducing the number of low birth weight infants. Consistent with previous studies (Gadomski et al. 2011), we also found a high prenatal smoking cessation rate of 68.3 % in participants with high session attendance. These participants consumed, on average, 63 % fewer cigarettes per day in the last 3 months of pregnancy compared with non-participants.

This study represents a first step in evaluating the effectiveness of the BMTF program with a focus on birth outcomes. The demonstrated effectiveness of the prenatal component of the program in reducing low birth weight may have potential public health and policy implications, given the increased morbidity and mortality and the substantial economic cost related to low birth weight infants (Goldenberg and Culhane 2007), and the fact that the majority of the program participants were Medicaid enrollees. However, the finding that only 36 % of the participants completed 3–4 prenatal cessation sessions highlights the challenge of maintaining the engagement of all participants over time. On the other hand, it indicates substantial potential for improvement of the program. As recognized by behavior economics, people are more likely to be motivated by immediate than delayed rewards (Loewenstein et al. 2007). This present-biased preference could be used to improve prenatal session attendance by offering incentives for attending prenatal sessions rather than waiting until postpartum, which might be more effective at promoting sustained cessation beyond pregnancy.

Several randomized controlled trials have consistently shown that use of financial incentives increased smoking cessation rates in pregnant women (Tappin et al. 2015; Higgins et al. 2012). However, fewer trials of financial incentives for smoking cessation in pregnant women have included birth outcomes as study endpoints. Some of these trials found a protective effect against low birth weight as seen in our study (Higgins et al. 2012), while others failed to show such an effect (Tappin et al. 2015). Similar to our finding, a potential protective effect on preterm birth was also suggested in some trials, but the results failed to reach statistical significance (Higgins et al. 2012). Although inadequate statistical power might be the main contributor, it is plausible that the discrepancy observed on low birth weight and preterm birth could be due to the observation that maternal smoking was more closely associated with fetal growth restriction than shortened gestation (Goldenberg and Culhane 2007).

Unlike randomized trials which investigated the efficacy of interventions in well-controlled settings, our study evaluated the effectiveness of a statewide, incentive-based smoking cessation program administered in “real” world community settings. The results of our study are generally in agreement with those of the trials, suggesting that it is feasible to translate trial findings on incentive-based cessation interventions into real world settings. Unique features of our study included a low-income study population from a state with a noticeably high rate of maternal smoking, the use of diaper vouchers as incentives with potentially added health benefits and greater public acceptability, and the finding on improving birth weight that could have important implications. However, as with many uncontrolled observational studies, our study has several important limitations, including a lack of ideal control group, reliance on birth certificate data on birth outcomes and covariates, the possibility of misclassification, and the possibility of residual confounding due to inaccurately measured or unmeasured covariates. The comparison group (non-participants) in our study presumably received standard care, which may have contributed to some of the observed variation in their smoking patterns across pregnancy. It is possible that non-participants might also have received other forms of interventions for smoking cessation which we could not account for. Nevertheless, the use of an active comparator would likely strengthen our conclusion.

In conclusion, our study found for the first time that successful participation in the BMTF program, an incentive-based smoking cessation program specifically designed for pregnant women, was associated with significantly decreased odds of having a low birth weight infant.
